# Enteroendocrine peptides regulate feeding behavior via controlling intestinal contraction of the silkworm *Bombyx mori*

**DOI:** 10.1371/journal.pone.0219050

**Published:** 2019-07-01

**Authors:** Sumihiro Matsumoto, Natsumaro Kutsuna, Ivana Daubnerová, Ladislav Roller, Dušan Žitňan, Hiromichi Nagasawa, Shinji Nagata

**Affiliations:** 1 Department of Integrated Biosciences, Graduate School of Frontier Sciences, The University of Tokyo, Chiba, Japan; 2 Institute of Zoology, Slovak Academy of Sciences, Bratislava, Slovakia; 3 Department of Applied Biological Chemistry, Graduate School of Agricultural and Life Sciences, The University of Tokyo, Tokyo, Japan; US Department of Agriculture, UNITED STATES

## Abstract

Our previous study demonstrated that predominant feeding inhibitory effects were found in the crude extracts of foregut and midgut of the silkworm *Bombyx mori* larvae. To address the entero-intestinal control crucial for the regulation of insect feeding behavior, the present study identified and functionally characterized feeding inhibitory peptides from the midgut of *B*. *mori* larvae. Purification and structural analyses revealed that the predominant inhibitory factors in the crude extracts were allatotropin (AT) and GSRYamide after its C-terminal sequence. *In situ* hybridization revealed that AT and GSRYamide were expressed in enteroendocrine cells in the posterior and anterior midgut, respectively. Receptor screening using Ca^2+^-imaging technique showed that the *B*. *mori* neuropeptide G protein-coupled receptor (BNGR)-A19 and -A22 acted as GSRYamide receptors and BNGR-A5 acted as an additional AT receptor. Expression analyses of these receptors and the results of the peristaltic motion assay indicated that these peptides participated in the regulation of intestinal contraction. Exposure of pharynx and ileum to AT and GSRYamide inhibited spontaneous contraction in *ad libitum*-fed larvae, while exposure of pharynx to GSRYamide did not inhibit contraction in non-fed larvae, indicating that the feeding state changed their sensitivity to inhibitory peptides. These different responses corresponded to different expression levels of their receptors in the pharynx. In addition, injection of AT and GSRYamide decreased esophageal contraction frequencies in the melamine-treated transparent larvae. These findings strongly suggest that these peptides exert feeding inhibitory effects by modulating intestinal contraction in response to their feeding state transition, eventually causing feeding termination.

## Introduction

Most phytophagous insects live on or around their preferred host plants [1]. Observations of these insects have demonstrated the presence of a regularly occurring pattern switching from a feeding mode to a quiescent mode, indicating unidentified endogenous regulatory mechanisms in both upregulation and downregulation of feeding motivation. The regulatory mechanisms in feeding motivation have been characterized by a number of physiological investigations, illuminating the importance of hemolymph factors corresponding to the nutritional status [2], eventually inducing in modifications in locomotor activities and neuronal signaling to maintain homeostasis [3, 4]. These biological homeostatic processes, including sequential feeding behavior and fluctuation in motivation, are known to be regulated by a number of neuropeptides in the central nervous system (CNS) and the peripheral organs [5, 6].

Among the feeding regulatory peptides in insects, several peptides are known as ‘brain-gut peptides’, which are expressed and produced in the brain and intestinal organs. Of the intestinal organs, the midgut possesses an important class of endocrine cells as well as the brain and CNS, where produce several neuropeptides, such as myosuppressin, neuropeptide F (NPF), tachykinin-related peptides (TRPs), and allatoregulatory peptides [7–10]. These peptides may regulate not only the physiological functions related to digestion [11] and intestinal lipid metabolisms [12] but also the intestinal contraction [13–15]. However, the current evidence is not adequate to evaluate the effects of “brain-gut peptides” on feeding regulation in insects. We have considered that the endogenous factors affecting the repetitive feeding state transition (feeding mode to quiescent mode) may be attributed to some of the feeding regulatory peptides [16]. In our previous study, significant feeding inhibitory factors that prolong the latency to the initiation of feeding (feeding-delaying activity) were observed in the extracts of the intestine (foregut, midgut) of *B*. *mori* larvae [17]. A previous biochemical study showed that *B*. *mori* RFamide has also similar feeding inhibitory activity [16]. In contrast, *B*. *mori* TRPs and short neuropeptide F (sNPF) -2 have feeding acceleratory effects that can shorten the latency to the initiation of feeding [16]. Although these reports demonstrate that endogenous endocrine factors regulate feeding motivation, little is known about their mode of action or how feeding state transition occurs.

In the present study, we purified and identified the predominant inhibitory factors from the midgut of *B*. *mori* larvae by assaying their behavioral activity on larval feeding. We further confirmed the inhibitory effects of these factors on intestinal contraction of the foregut and hindgut, illuminating a possible model for a feeding regulatory mechanism mediated by enteroendocrine factors that modulate intestinal contraction.

## Materials and methods

### Animals

Eggs of the silkworm, *Bombyx mori* (a hybrid strain, Kinshu × Showa), were purchased from a silkworm egg-producing company, Ueda Sanshu Ltd. (Ueda, Japan). Larvae were reared in plastic containers at 26 ± 1°C at 70 ± 10% relative humidity, and under 16 L:8 D lighting conditions. Larvae were fed a fresh artificial diet of Silkmate 2S purchased from Nippon Nosan Co. Ltd. (Yokohama, Japan).

### Chemicals and reagents

The chemicals and reagents used in this study were purchased from Nacalai-tesque (Osaka, Japan). HPLC reagents (acetonitrile and trifluoroacetic acid [TFA]) were purchased from Kanto Kagaku (Tokyo, Japan). Fmoc derivatives of the amino acids were purchased from Watanabe Kagaku (Hiroshima, Japan).

### Purification of biologically feeding inhibitory peptides

Midguts isolated from 500 2-day-old fifth-instar *B*. *mori* larvae fed *ad libitum* were homogenized in 90% methanol containing 1.0% acetic acid on ice. After centrifugation at 2,300 × *g* for 15 min, the supernatant was evaporated to dryness, redissolved in a 0.1% TFA aqueous solution. The resulting solution was applied to a Sep-Pak C18 Cartridge (Waters, Milford, MA, USA) and the crude peptide fraction was collected as the fraction eluted with 60% acetonitrile containing 0.1% TFA after washing the un-adsorbed substances with a 0.1% TFA aqueous solution followed by 10% acetonitrile containing 0.1% TFA. The resulting eluent was subjected to reversed-phase high-performance liquid chromatography (RP-HPLC) on a UG 120Å ODS column (10 mm i.d. × 250 mm, Senshu Kagaku, Tokyo, Japan). The elution was performed with a linear gradient of 10–60% acetonitrile containing 0.1% TFA over 50 min at a flow rate of 2.0 mL/min. The elution was monitored by the absorbance at 280 nm. The biologically active fractions were evaporated to dryness, redissolved in a 0.1% TFA aqueous solution and then subjected to the next step of RP-HPLC using a PEGASIL ODS column (4.6 mm i.d. × 250 mm, Senshu Kagaku) in a linear gradient of 10–60% acetonitrile containing 0.1% TFA over 50 min at a flow rate of 1.0 mL/min. The elution was monitored by the absorbance at 280 nm. The purifications according to biological activities possibly until obtaining an isolated peptide were carried out by RP-HPLC using different of linear gradient program conditions monitored by absorbance at 225 nm. Mass spectra of the substances in the biologically active fractions were measured using a matrix-assisted laser desorption ionization time-of-flight (MALDI-TOF) mass spectrometer.

### MALDI-TOF mass spectrometry

Molecular mass of peptides was measured by a MALDI-TOF mass spectrometer Voyager-DETM STR (Applied Biosystems, Foster City, CA, USA) in a positive ion mode. Samples were prepared by mixing with the supernatant of α-cyano-4-hydroxycinnamic acid saturated in 60% acetonitrile as a matrix at 1:1 ratio.

### Feeding behavioral assay

A part of the eluents of the fractions from RP-HPLC as well as synthetic peptide solutions was evaporated to dryness and dissolved in 100 μL distilled water just prior to the bioassay. The feeding cycles of 2-day-old fifth-instar *B*. *mori* larvae were synchronized by diet deprivation for 16 h. After diet deprivation, the larvae were anesthetized by submerging in icy water for 15 min. Then, 100 μL sample was injected dorsolaterally into the hemolymph. After injection, the larvae were placed in front of artificial diet blocks, and then the latency to their first bites as the initiation of feeding (first bite) after sample-injection was measured as reported previously [16]. Statistical analysis was performed using a one-way analysis of variance (one-way ANOVA), then a post hoc Dunnett’s test. Reproducibility was confirmed at least twice by assays using different populations of larvae.

### Amino acid sequence analysis and homology search

The N-terminal amino acid sequence of the purified peptide was analyzed by a protein sequencer Procise^TM^ cLC (Applied Biosystems) in pulsed-liquid mode. A Basic Local Alignment Search Tool (BLAST) search for the entire sequences of the obtained partial sequences was performed using the *B*. *mori* databases Silk Base (http://silkbase.ab.a.u-tokyo.ac.jp/) and KAIKO BLAST (http://kaikoblast.dna.affrc.go.jp/). A homology search of GSRYa-1 and -2 (S3 Fig) was performed by using GenBank (http://www.ncbi.nlm.nih.gov/genbank/) and BLAST searches (blastn, tblastn, and Blastp).

### Preparation of synthetic peptides

Peptides were synthesized by the standard solid-phase methodology (Fmoc procedure) on an AAPPTEC APEX 396-SC synthesizer (AAPPTEC, Louisville, KY, USA). The resulting crude synthetic peptides after deprotection were purified using a Sep-Pak C18 Cartridge (Waters) and by RP-HPLC on a PEGASIL-300 ODS column (10 mm i.d. × 250 mm or 4.6 mm i.d. × 250 mm, Senshu Kagaku) using the identical linear gradient condition as that used for purification from midgut according to MALDI-TOF mass analyses. The synthetic peptides solutions were evaporated to dryness and dissolved in distilled water or *B*. *mori* Ringer’s solution (110 mM KCl, 4 mM NaCl, 15 mM MgCl_2_, 4 mM CaCl_2_, 5 mM KH_2_PO_4_, pH 6.5) for biological assay. The concentration of eluted synthetic peptide solutions was adjusted after measuring the peptide solution by Bradford protein assay [18]. The purities of the all used peptides in the experiments were confirmed to be higher than 97.0%.

### Reverse transcriptase polymerase chain reaction (RT-PCR)

Total RNA was extracted from the isolated tissues of 2-day-old fifth-instar larvae fed *ad libitum* (brain, CNS, foregut, anterior, middle and posterior midgut, hindgut, Malpighian tubules, fat body, silk gland, testis, ovary, and hemocytes) using TRIzol Reagent (Invitrogen, Carlsbad, CA, USA) according to the manufacturer’s instructions. The extracted total RNA was treated with DNase I (TaKaRa Bio, Shiga, Japan) at 37°C for 1 h. cDNA was synthesized using total RNA with oligo (dT) and Superscript III Reverse Transcriptase (Invitrogen). RT-PCR was performed using the resulting cDNA as a template and the following primer sets: AT-Fw (5´-AGCAGGCAGTCTCACGAGTT-3´) and AT-Rv (5´-CGCAAACCGATTTTAACAGA-3´), GSRYamide-Fw (5´-GTGCGTGCGTGTACTACGAT-3´) and GSRYamide-Rv (5´-CATTGGCATGGCAAAGTCTA-3´), *B*. *mori* neuropeptide G protein-coupled receptor (BNGR)-A5-Fw (5´-TACGTGTTCCCTCAGCCCTA-3´) and BNGR-A5-Rv (5´-TTTGCACTTGAAGCACCACG-3´), BNGR-A16-Fw (5´-TGGAGCTCCAACGGAATTCC-3´) and BNGR-A16-Rv (5´-GTGCTGGTATGTCCTGGCTT-3´), BNGR-A19-Fw (5´-AGAATGGGACCACGTGGAAC-3´) and BNGR-A19-Rv (5´-TGAAGCCGCGTCGATATCTC-3´), BNGR-A22-Fw (5´-AAGCCTCGACTTGGGAAAGG-3´) and BNGR-A22-Rv (5´-TGACTCGCGAACCAAACGTA-3´). Ribosomal protein L3 (rpL3) was used for experimental control. Utilized primers were rpL3-Fw (5´-TGGCACACAAAGAAGCTACCC-3´) and rpL3-Rv (5´-TGACCAGCACGAGCTACAGTG-3´). Ex-Taq polymerase (TaKaRa Bio) was used for PCR. The PCR program was as follows. Amplification: 30 cycles of 15 s at 94°C (3 min for the first cycle), 30 s at 55°C, and 45 s at 72°C. The PCR products were electrophoresed on a 1.5% agarose gel and stained with ethidium bromide. The PCR products were subcloned into pGEM-T Easy vectors (Promega, Madison, WI, USA) and were confirmed by sequencing on an ABI PRIZM 310 Genetic Analyzer (Applied Biosystems) using a Big Dye Terminator Ver.3.1 Cycle Sequencing Kit (Applied Biosystems).

### *In situ* hybridization

Whole mount *in situ* hybridization was performed as described by Roller et al. [19] and Bednár et al. 2017 [20]. The digoxigenin-labeled single-stranded DNA probes were synthesized by asymmetric PCR. The sense and antisense probes of AT and GSRYamide were synthesized using the primer sets: AT-Fw (5´-AGCAGGCAGTCTCACGAGTT-3´) and AT-Rv (5´-CGGGTTGTTGAGAACCTCAT-3´), GSRYamide-Fw (5´-GTGCGTGCGTGTACTACGAT-3´) and GSRYamide-Rv (5´-CATTGGCATGGCAAAGTCTA-3´). The probe size of AT and GSRYamide were 360 nt and 521 nt, respectively.

The midgut from fourth-instar larvae was isolated and fixed overnight at 4°C in 4% paraformaldehyde (PFA) in phosphate buffered saline (PBS), and washed with PBS containing 0.1% Tween 20 (PBST). The samples were digested with proteinase K (50 μg /mL PBST) for 10 min, washed with Glycine-PBST (2 mg Glycine/1 mL PBST), postfixed with 4% PFA for 1 h and washed with PBST. Prehybridization was performed in the hybridization solution (HS; 50% formamide, 5 × Saline sodium citrate, 100 mg/mL salmon testes DNA, 0.1% Tween 20, 50 mg/mL heparin, RNase-free deionized water) at 48°C for 1–2 h. The tissues were incubated in HS containing the DNA probe (1 volume of probe per 9 volumes of HS) specific for the AT and GSRYamide gene transcripts at 48°C for 20–24 h. After hybridization, the tissues were washed with HS at 48°C overnight and briefly with a mixture of HS-PBST (1:1) and PBST at room temperature. They were then blocked with 1% bovine serum albumin (BSA; Sigma-Aldrich Co. LLC., St. Louis, MO, USA) in PBST for 10 min, incubated with an alkaline phosphatase (AP)-labelled anti-digoxigenin antibody (1:1000; Roche, Mannheim, Germany) overnight at 4°C, and stained with a Nitroblue tetrazolium and 5-Bromo-4-chloro-3-indolyl phosphate solution (NBT-BCIP, Roche) diluted with 1:50 in AP buffer (100 mM Tris, 50 mM MgCl_2_, 100 mM NaCl, 0.1% Tween 20, pH 9.5). After color development, they were washed with PBST, then mounted in glycerol. Negative controls were performed using a specific sense probe. Staining was detected using a LEICA M205FA stereomicroscope (Leica Microsystems, Wetzlar, Germany) and a Nikon Eclipse 600 microscope with Nomarski differential interference contrast optics and photographed using an attached Nikon Coolpix 990 digital camera (Nikon, Tokyo, Japan). Images are presented as maximum projection of Z-stacks. Stacking was performed using CombineZP (shareware by Alan Hadley).

### Phylogenetic analysis

A phylogenetic tree was generated using CLUSTAL W by neighbor-joining method. Bootstrap of 1,000 replications was conducted to assess the relationships. The deduced amino acid sequences of 36 class A BNGRs; BNGR-A1 (NP_001127736), BNGR-A2 (NP_001127737), BNGR-A3 (NP_001127737), BNGR-A4 (NP_001127739), BNGR-A5 (NP_001127740), BNGR-A6-A (NP_001127741), BNGR-A6-B (NP_001165737), BNGR-A7 (NP_001127742), BNGR-A8 (NP_001127743), BNGR-A9 (NP_001127744), BNGR-A10 (NP_001127707), BNGR-A11 (NP_001127708), BNGR-A12 (NP_001127709), BNGR-A13 (NP_001127710), BNGR-A14 (NP_001127711), BNGR-A15 (NP_001127712), BNGR-A16 (NP_001127714), BNGR-A17 (NP_001127715), BNGR-A18 (NP_001127716), BNGR-A19 (NP_001127720), BNGR-A20 (NP_001127718), BNGR-A21 (NP_001127719), BNGR-A22 (NP_001127717), BNGR-A23 (NP_ 001127721), BNGR-A24 (NP_001127722), BNGR-A25 (NP_001127723), BNGR-A26 (NP_001127724), BNGR-A27 (NP_001127725), BNGR-A28 (NP_001127726), BNGR-A29 (NP_001127745), BNGR-A30 (NP_001127746), BNGR-A31 (NP_001127747), BNGR-A32 (NP_001127748), BNGR-A33 (NP_0011227749), BNGR-A34 (NP_001127750), BNGR-A35 (NP_001127751), *B*. *mori* diuretic hormone receptor variant X2 (DHR) (XM_021352745.1), *Drosophila melanogaster* myosuppressin receptor 1 isoform A (D-MsR1) (NP_647713), *D*. *melanogaster* myosuppressin receptor 2 isoform A (D-MsR2) (NP_647711), *D*. *melanogaster* sNPF receptor isoform A (D-sNPFR) (NP_524176), *D*. *melanogaster* tachykinin-like receptor at 86C, isoform A (D-TkR86C) (NP_524304), *D*. *melanogaster* tachykinin-like receptor at 99D, isoform A (D-TkR99D) (NP_524556), *Tribolium castaneum* RYamide receptor (T-RYaR) (NP_001280539), and *D*. *melanogaster* RYamide receptor isoform A (D-RYaR) (NP_524525) were obtained from GenBank by BLAST searches (blastn, tblastn and Blastp). *B*. *mori* DHR was used as an outgroup.

### Ca^2+^-imaging assay of BNGR-A19, -A22, -A23, -A24, -A32, and -A33

Human embryonic kidney (HEK) 293 cells were cultured in Dulbecco’s modified Eagle’s medium (DMEM; Wako Pure Chemical Industries, Ltd., Osaka, Japan) supplemented with 10% fetal bovine serum (Thermo Fisher Scientific Inc., Waltham, MA, USA) at 37°C in a humidified atmosphere containing 5% CO_2_. HEK293 cells seeded on a 35-mm glass-based dish (Iwaki, Tokyo, Japan) were co-transfected with pME18S plasmids carrying BNGR cDNAs and the promiscuous G protein alpha subunit (Gα_15_) using Lipofectamine LTX with Plus Reagent (Thermo Fisher Scientific Inc.) according to the manufacturer’s protocol.

The Ca^2+^ indicator solution consisting of 2 μM Fluo-4 AM (Thermo Fisher Scientific Inc.) and 0.01% pluronic-F 127 (Sigma-Aldrich Co. LLC.) in Opti-MEM (Thermo Fisher Scientific Inc.) was added to the cells after 24 h transfection at 37°C for 30 min in the dark. After incubation, the cells were washed twice with Ringer’s solution (140 mM NaCl, 5.6 mM KCl, 2.0 mM CaCl_2_, 2.0 mM MgCl_2_, 9.4 mM D-glucose, 1.25 mM KH_2_PO_4_, and 5 mM HEPES, pH 7.4), and then the Ringer’s solution was added. The Ca^2+^-imaging assays were performed within 2 h after replacement with Ringer’s solution. Using a confocal laser-scanning microscope LSM5 Exciter (Zeiss, Oberkochen, Germany), the intensity of fluorescence in Fluo-4-loaded cells at 518 nm by excitation at 488 nm was recorded during measurement for 1400 s with the following supplements in the Ringer’s solution; synthetic peptides (10^−10^–10^−5^ M final concentration) at 180, 360, 540, 720, 900, 1080, and 100 μM ATP (a ligand for an endogenous ATP receptor) at 1260 s as a positive experimental control for robust intracellular Ca^2+^ increase. Data were calculated by the responding fluorescent intensities of 20 cells which did not respond to the exposure to Ringer’s solution (at 0 s) and showed responses to exposure to the ATP solution, as the relative fluorescent intensity (0%, the minimum intensity; 100%, the maximum intensity during monitoring).

### Ca^2+^-imaging assay of BNGR-A5 and -A16

Activity of the BNGR-A5 and -16 receptors were analyzed using Chinese hamster ovary (CHO) cells transiently transfected with plasmids for expression of each BNGR, calcium-sensitive reporter aequorin and chimeric G protein that couple with receptor activation to an intracellular Ca^2+^-increase independent of the endogenous receptor signaling cascade. The entire open reading frames of BNGR-A5 and -16 were cloned into pcDNA3.1(+) vector (Invitrogen) with a Kozak translation initiation sequence incorporated at 5´end. Plasmids for codon optimized aequorin and chimeric G proteins were used as described previously [21–23].

CHO cells were maintained at 37°C in a humidified atmosphere of 5% CO_2_ in DMEM Nutrient Mixture F-12 Ham (DMEM/F12) with L-glutamine, 15 mM HEPES, sodium bicarbonate and phenol red (Sigma-Aldrich Co. LLC.) enriched with 10% heat-inactivated fetal bovine serum (Sigma-Aldrich Co. LLC.), 100 IU/mL penicillin and 100 μg/mL streptomycin (Life Technologies, Carlsbad, CA, USA). Approximately 24–30 hours before transfection, we seeded appropriate number of CHO cells in 10 mL of complete growth medium in 10 cm Petri dishes to achieve 60–80% confluency at the time of transfection. Each receptor assay was performed using transfected cells from two Petri dishes. For cell transfection, we prepared 20 μg DNA directly into 950 μL of serum- and antibiotic-free medium and incubated the mixture for 5 minutes at room temperature. Thereafter, we added 30 μL of warm FuGene HD (Promega) to the tube and incubated the transfection reagent to make DNA complex at room temperature for 25 min. We then removed the cell medium from Petri dishes, followed by covered the cells with 10 mL of fresh antibiotic-free culture medium and added the transfection mixture in a dropwise manner. Cells were allowed to grow for 24–48 hours (37°C, 5% CO_2_) and used for the assay.

The transfected cells were detached using PBS supplemented with 0.2% EDTA and rinsed of the culture plates with the assay medium—phenol red free DMEM/F12 with l-glutamine and 15 mM HEPES (PAN Biotech, Wimborne, Dorset, UK), 0.1% BSA (Sigma-Aldrich Co. LLC.) and 1% penicillin/streptomycin (Life Technologies). Then we centrifuged cells at 1000 rpm for 3 min, aspirated the medium and resuspended the cell pellet in the assay medium (10 mL). We repeated centrifugation step (1000 rpm, 3 min), resuspended the pellet in the assay medium (5 mL) and coelenterazine H (Promega) was added at a final concentration of 5 μM. Cells were then maintained in suspension with a stirrer at room temperature for 2.5–3 h in the dark to reconstitute the active holoenzyme aequorin. 30 min prior to the measurement cells were diluted threefold using the same BSA medium and 50 μL of cell suspension was injected into each well of a 96-well plate filled with 50 μL of tested compounds dissolved in the assay medium. Various peptide concentrations were loaded in triplicates and wells containing a negative control (assay medium without ligands) was included in each row to correct cell response of each well in the same row. Emitted luminescence signal corresponding to the ligand-induced intracellular Ca^2+^ release was recorded for 20 s using the GloMax-Multi+ Detection System (Promega). Wells containing ATP at a final concentration of 50 μM served as a positive control and 100 μM digitonin was used to measure the total receptor-independent cellular Ca^2+^-response. Each experiment was repeated three times for data analysis. Calculations and further analysis of the output data were done in Excel (Microsoft, Redmond, WA, USA) and GraphPad Prism 6 software.

### Observations of spontaneous contractions of the foregut and hindgut in dissected open larvae

The opened head and abdominal portion of 2-day-old fifth-instar larvae (fed *ad libitum* and non-fed for 16 h) were washed twice with *B*. *mori* Ringer’s solution and incubated for 3 min at 27°C to stabilize the movement. After incubation, the contractile movements of the larval foregut and hindgut [24], particularly the spontaneous contraction of the pharynx and ileum (predominantly contracting area in hindgut) were observed and recorded using a digital microscope USB2.0 DigiScope II v2^TM^ (CHRONOS, Taipei, Taiwan). 100 μL of *B*. *mori* Ringer’s solution was added to the examined gut at 60 s after starting recording. Furthermore, 100 μL of *B*. *mori* Ringer’s solution (vehicle control) and 100 μL peptide-containing solution (10^−9^–10^−5^ M of peptides dissolved in *B*. *mori* Ringer’s solution) were exposed over gut at 120 s after a recording start. The contractile movements were analyzed using ImageJ software (https://imagej.net/Fiji) with the Optic flow plug-in (Gaussian window MSE). The direction of contractile movement was represented by the color and arrows described in the ‘Direction’ map. The position of the lines used for the kymograph analyses (x-y, z-w) were determined by Optic flow analyses. The waveform of the kymographs, corresponding to the visible contractions in the recorded movies, were quantified and converted to myograms. The number of contractions in 60 s was defined as the ‘contraction frequency’. Invisible contractions in the recorded movie and extremely small amplitudes in the myograms were considered as no significant contractions. The evaluation of visible contraction was defined by giving a threshold at the half length of the observed amplitude. The effect of synthetic peptides was represented by the ratio of the ‘contraction frequency’ at 60–120 s to 120–180 s (%) due to the extendedly various frequencies by individuality; the pharynx (30-40/60s) and hindgut (20-40/60s). This methodology by kymograph is substantially corresponding to the previously used assays for intestinal contraction [25–27]. Statistical analyses were performed compared with the vehicle control by one-way ANOVA, then a post hoc Dunnett’s test. Reproducibility was confirmed at least twice by assays using different populations of larvae.

### Quantitative polymerase chain reaction (Q-PCR)

Total RNA was extracted from the isolated tissues (pharynx, esophagus, anterior and posterior midgut, pylorus-ileum, colon, and rectum) of 2-day-old fifth-instar larvae fed *ad libitum* and non-fed for 16 h. The first-strand cDNA was used as a template and each amplification was primed by a pair of primers: BNGR-A19-Fw (5´-ATGAAGAGTGGGCTGCATGG-3´) and BNGR-A19-Rv (5´-AATGTGACATCGCCAGCCA-3´). Ribosomal protein L3 (rpL3), Glyceraldehyde-3-phosphate dehydrogenase (GAPDH) and α-tubulin were used for experimental control. Utilized primers were rpL3-Fw (5´-TCGTCCAAGTTCGGTCATGG-3´) and rpL3-Rv (5´-ACCCATGAATGCAGCCTTGT-3´), GAPDH-Fw (5´-TTCCTGCCTCTACTGGTGCT-3´) and GAPDH-Rv (5´-CCATTCCAGTCAGCTTGCCA-3´), α-tubulin-Fw (5´-CGCACTGGCACATACAGACA-3´) and α-tubulin-Rv (5´-TTGTTGGCCGCATCTTCCTT-3´). Q-PCR was performed using a Thermal Cycler Dice Real-Time System (TaKaRa Bio), and THUNDERBIRD SYBR qPCR Mix (TOYOBO, Osaka, Japan) and 0.4 μM of each primer. The thermal cycling conditions were as follows: initial denaturation at 95°C for 1 min and then 45 cycles of 95°C, 10 s; and 60°C, 30 s. The single targeted Q-PCR product was confirmed by generating a melt curve for all reactions. Data were analyzed using the Thermal Cycler Dice Real-Time System Single Software Version 5.11 (TaKaRa Bio). The results are represented as a ratio of BNGR-A19 and rpL3 by comparative CT method. Statistical analysis was performed to compare with the larvae fed *ad libitum* in each tissue by Student’s *t*-test.

### Observations of the esophageal contraction in intact larvae

Transparent larvae were prepared by feeding an artificial diet containing 0.05% melamine (Wako Pure Chemical Industries) starting from 0-day-old fifth-instar larvae. Melamine-feeding enhanced the excretion of uric acid and rendered the larval integument transparent as previously reported [28]. The visible effect took place in 2-day-old fifth-instar larvae compared to normally fed larvae. The feeding behavioral assay of transparent larva was performed in the same way as that of normal larvae. Each transparent larva was surrounded with a soft sponge and held with both sides by two slide glasses. Transmitted light enabled to illuminate the shape and movement of the shadowed esophagus portion. The contraction of foregut, particularly ‘forward contraction wave’ and ‘peristaltic squeeze’ at the esophagus [24] were observed using a digital microscope to measure the number of contractions per minute. The ‘peristaltic squeezes’ at 8 h and 16 h in non-fed larvae were unmeasurable because of the limit of detection in our observation using digital microscope. 100 μL of distilled water (vehicle control) and 100 μL peptide-containing solution (10^−7^–10^−4^ M of peptides dissolved in distilled water) were injected dorsolaterally into the hemolymph. After injection, the numbers of contractions per minute of the esophagus were counted from the data of 3 min observation. Statistical analysis was performed using a one-way ANOVA, then a post hoc Dunnett’s test. Reproducibility was confirmed at least twice by assay using different populations of larvae.

## Results

### Purification and identification of feeding inhibitory factors from the midgut of *B*. *mori* larvae

**Fig 1:**
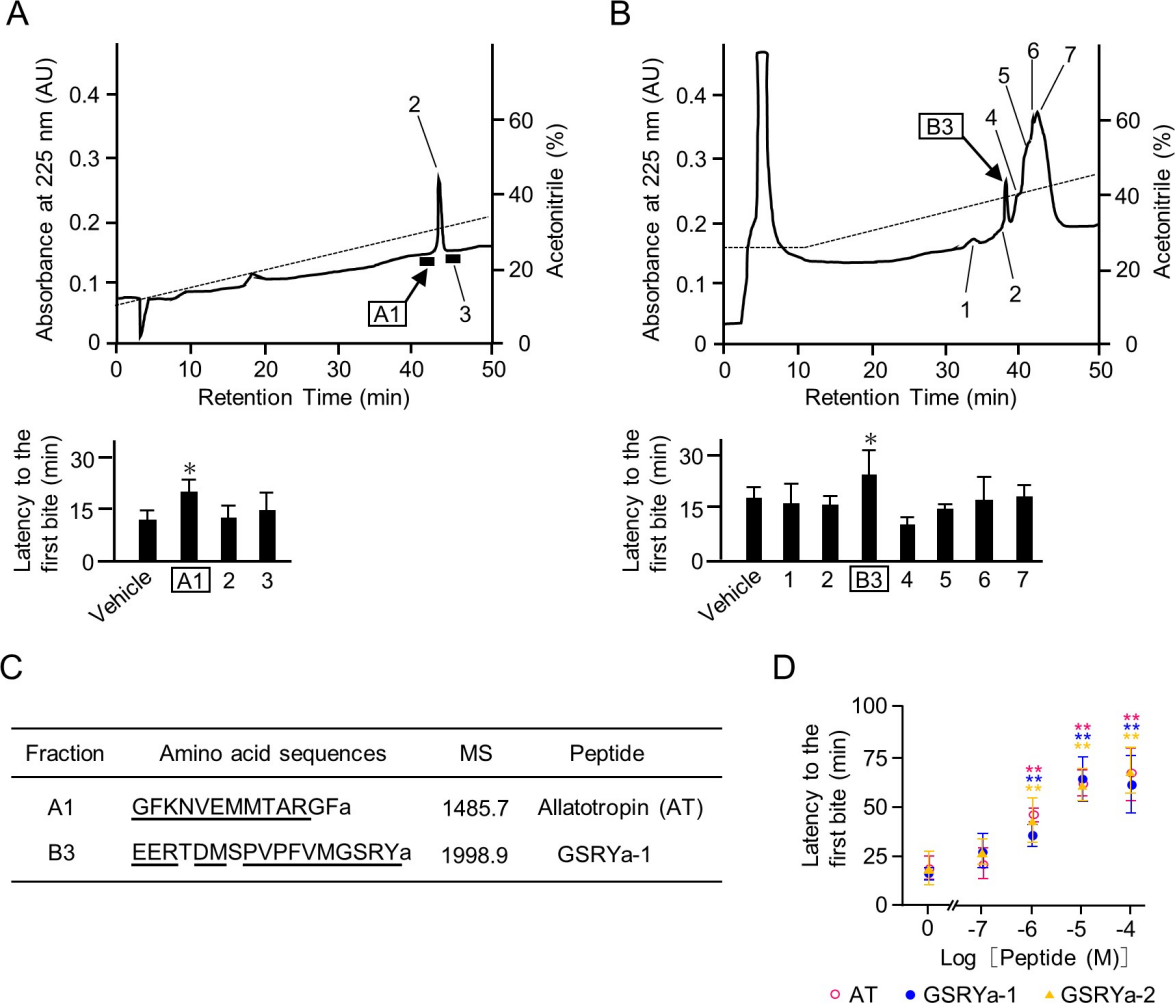
Purification and identification of feeding inhibitory factors from the midgut of *B*. *mori* larvae. (A) and (B) RP-HPLC profiles of the final purification step of the midgut extracts (upper) and their feeding inhibitory activities (lower). Upper; The dashed lines represent the acetonitrile concentrations. The numbered areas and peaks indicate the fractions used for behavioral bioassays. The injected dose of each sample was 20 midgut equivalents of the fifth-instar larvae. Lower; Bars represent means of the latency to the first bite. An asterisk indicates a significant difference compared to vehicle-injected larvae by one-way ANOVA (*P* < 0.05). Data represent means + S.D. (n = 3). (C) Amino acid sequences of feeding inhibitory factors in the A1 and B3 fractions. N-terminal sequences identified by the protein sequencer are underlined. ‘a’ indicates C-terminal amidation. Masses are given in Dalton (Da) and represent monoisotopic uncharged mass (MS). (D) Dose-dependent effects of synthetic AT and GSRYa-1 and -2 on the latency to the first bite. Asterisks (**) indicate significant differences compared with vehicle-injected larvae by one-way ANOVA (*P* < 0.01). Data represent means ± S.D. (n = 3).

To identify the feeding inhibitory factors in *B*. *mori* larvae, we purified these factors into two fractions at the sixth purification step of RP-HPLC according to their biological activities, which significantly prolonged the latency to the first bite after sample injection compared with those by vehicle injection (A1 and B3 in Fig 1A and 1B). The two purified biologically active fractions were then sequenced, consequently providing a single predominant N-terminal sequence in each fraction. BLAST search of the obtained N-terminal sequences in the *B*. *mori* databases and MALDI-TOF mass analyses (S1 Fig) revealed that the predominant peptides were allatotropin (AT) in fraction A1 and a peptide composed of 17 amino acids in fraction B3 (Fig 1C). The latter peptide was an ortholog of the recently discovered insect RY-amide [29, 30], and was accompanied by its paralogous peptide in the precursor peptide (S2 Fig); we then designated them as GSRYamides (GSRYa-1 and -2) after their common C-terminal amino acid sequences. The feeding inhibitory activities of these three peptides were confirmed by the use of synthetic peptides. Injection of AT and GSRYa-1 and -2 prolonged the latency to the first bite in a dose-dependent manner (ED_50_ = 1.0 × 10^−6^ M, 1.3 × 10^−6^ M, and 1.0 × 10^−6^ M, respectively) (Fig 1D). These data indicated that AT and GSRYa-1 and -2 are the predominant inhibitory peptides in the midgut of *B*. *mori* larvae. In addition, we further investigated the effects of these peptides on intestinal motility.

### Spatial expression analyses of AT, GSRYamide, and their cellular distribution of enteroendocrine cells (EEs) in the larval midgut

**Fig 2:**
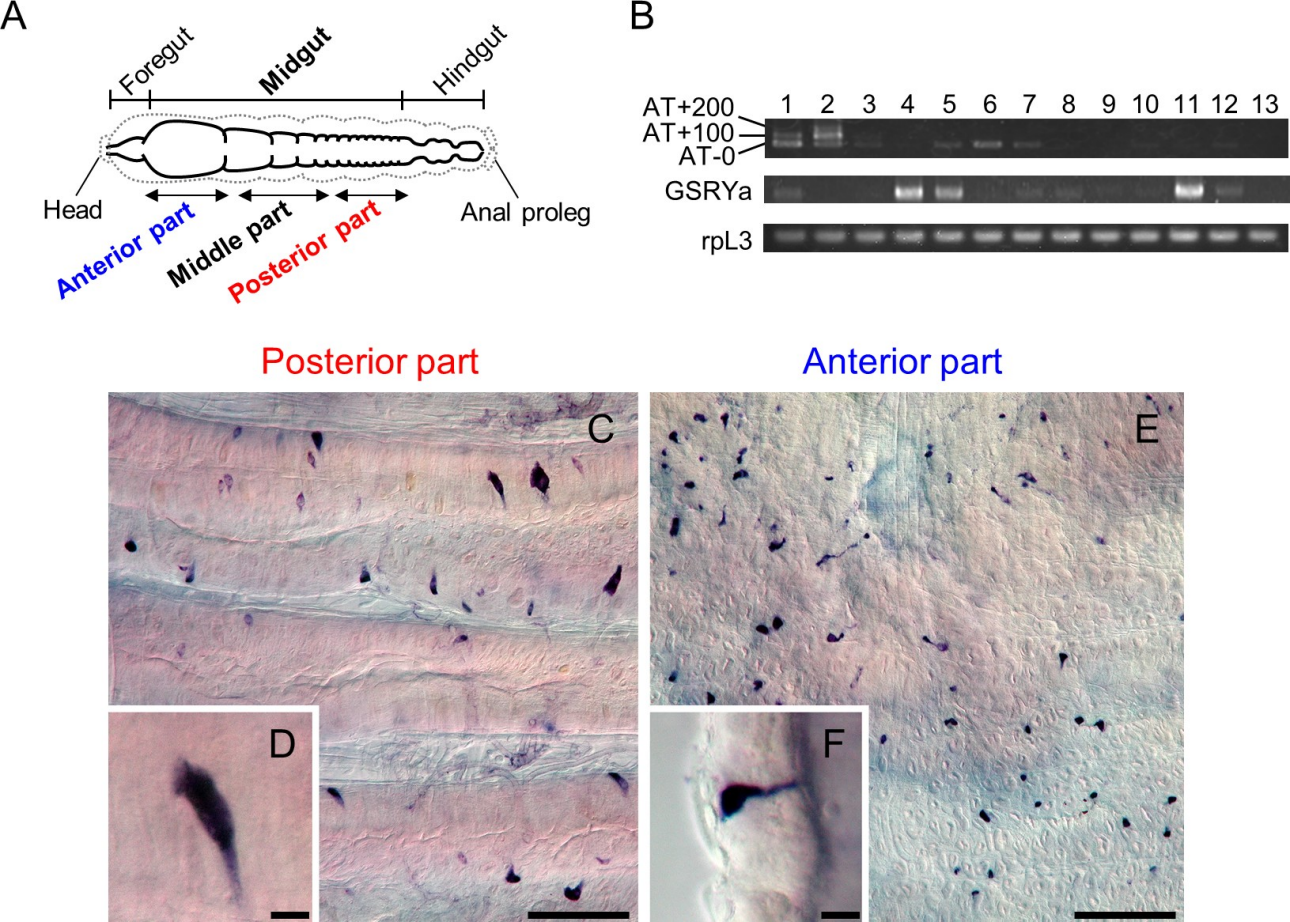
Expression analyses of AT and GSRYamide and the cellular distributions of EEs in the midgut. (A) A schematic diagram of *B*. *mori* larval intestine (three parts of the midgut). Dashed line indicates the outline of the larval body. (B) RT-PCR analyses of AT and GSRYamide (GSRYa). Ribosomal protein L3 (rpL3) was used as an experimental control. Brain (lane 1), CNS (lane 2), foregut (lane 3), anterior, middle and posterior midgut (lanes 4, 5 and 6), hindgut (lane 7), Malpighian tubules (lane 8), fat body (lane 9), silk gland (lane 10), testis (lane 11), ovary (lane 12), and hemocyte (lane 13). (C) *In situ* hybridization of AT. AT-expressing EEs were scattered in the posterior midgut of fourth-instar larvae. (D) A zoomed-in image of a pyramid-shaped EE expressing AT. (E) *In situ* hybridization of GSRYamide. GSRYamide-expressing EEs were scattered in the anterior midgut of fourth-instar larvae. (F) A zoomed-in image of bottle-shaped EE expressing GSRYamide. Scale bars = 100 μm in (C), (E) and 10 μm in (D), (F).

We next analyzed the spatial expression pattern of the identified feeding inhibitory peptides (AT and GSRYamide) in the *B*. *mori* larvae. RT-PCR revealed that AT was expressed in the middle and posterior midgut, while GSRYamide was strongly expressed in the anterior and middle midgut as well as in the reproductive organs (testis and ovary) (Fig 2A and 2B). In addition, expression of AT and GSRYamide was observed in the brain. As previously reported [31], PCR products differing by approximately 100 bp due to alternatively spliced variants of AT and AT-like peptides have been observed in the brain and CNS. In the case of AT expression in the midgut, we observed only the transcript for AT-0 encoding one of the AT sequence (Fig 2B).

*In situ* hybridization revealed that AT-expressing EEs were located in the posterior midgut (Fig 2C), while GSRYamide-expressing EEs were present in the anterior midgut (Fig 2E). Most of the AT-expressing cells (30–35 μm) were pyramid-shaped EEs, whose broad ends might be in contact with the basal lamina (Fig 2D). In contrast, most GSRYamide-expressing cells (20–25 μm) [19] were bottle-shaped EEs, having a broad basal region and cytoplasmic apical processes (Fig 2F). These data indicated that the AT and GSRYamide enteroendocrine peptides were expressed and produced in different regions and distinctive types of EEs in the midgut.

### Expression of AT and GSRYa-1 and -2 receptors in the intestinal organs

**Fig 3:**
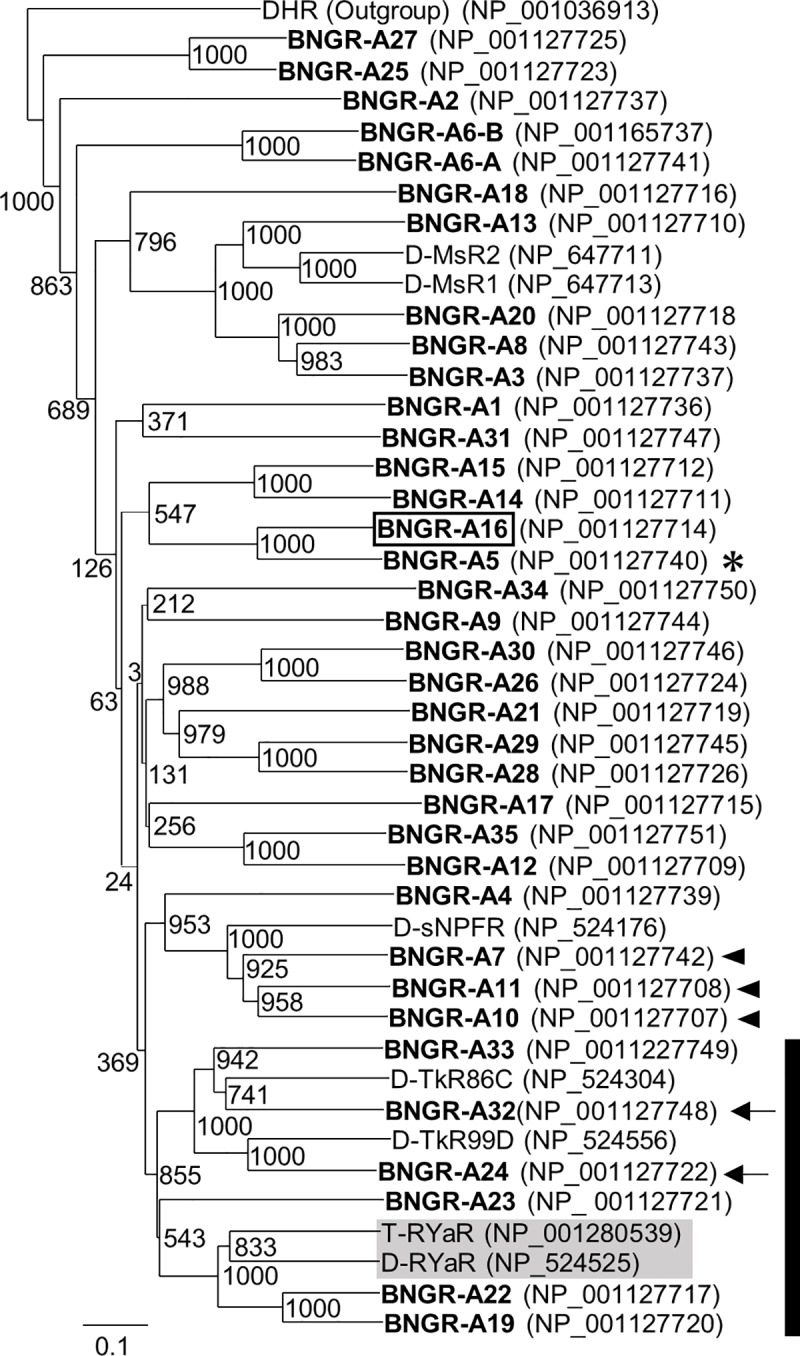
Phylogenetic tree of neuropeptide G protein-coupled receptors in *B*. *mori* and other insects. Phylogenetic tree of BNGRs (bolded) and *D*. *melanogaster* and *T*. *castaneum* G protein-coupled receptors; D-MsR1, D-MsR2, D-sNPFR, D-TkR86C, D-TkR99D, D-RYaR, and T-RYaR. Deorphanized BNGRs are indicated in parentheses; Bm-ITPR (*B*. *mori* ion transport peptide receptor) [34], Bm-ETHR (*B*. *mori* ecdysis-triggering hormone receptor) [32], Bm-ATSCR (*B*. *mori* allatostatin C receptor) [32], Bm-ATR (*B*. *mori* allatotropin receptor) [32], Bm-CRZR (*B*. *mori* corazonin receptor) [35], Bm-AKHR (*B*. *mori* adipokinetic hormone receptor) [36], Bm-NPFR (*B*. *mori* neuropeptide F receptor) [37], Bm-sNPFR (*B*. *mori* short neuropeptide F receptor) [32, 38], Bm-NTLR (*B*. *mori* natalisin receptor) [39], Bm-TRPR (*B*. *mori* tachykinin-related peptide receptor) [40], Bm-ITPLR (*B*. *mori* ion transport peptide-like receptor) [34]. BNGR-A5 (an arrow) was newly validated as another AT receptor. *B*. *mori* DHR was used as an outgroup. BNGR-A19, -A22, -A23, -A24, -A32, and -A33 in the clade (black bar) including T-RYaR and D-RYaR (gray-boxed) [29, 41] were screened as potential GSRYa-1 and -2 receptors.

**Fig 4:**
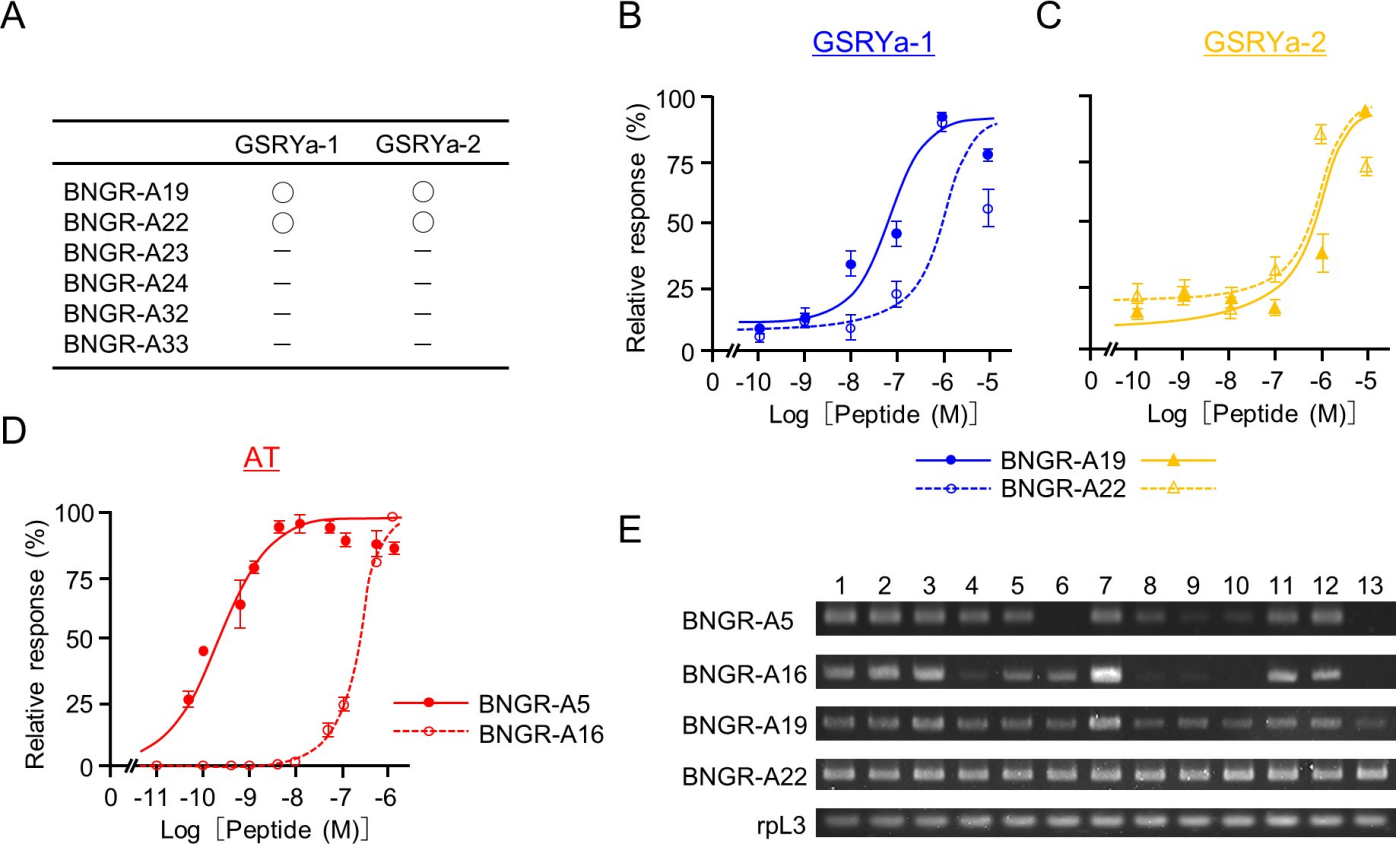
Screening for AT and GSRYa-1 and -2 receptors and expression analyses of BNGRs. (A) Ca^2+^-imaging analyses of six BNGRs (BNGR-A19, -A22, -A23, -A24, -A32, and -A33) to GSRYa-1 and -2 using HEK293 cells. Circles of BNGR-A19 and -A22 indicate intracellular Ca^2+^ increase following exposure to GSRYa-1 and -2 in a dose-dependent manner. Dashes of BNGR-A23, -A24, -A32, and -A33 indicate no response in all doses. (B) and (C) Dose-response analyses of BNGR-A19 and -A22 to synthetic GSRYa-1 (closed and open circles) and GSRYa-2 (closed and open triangles). Data are shown as relative fluorescent intensities (mean ± S.E.). (D) The dose-response analyses of BNGR-A5 and -A16 to synthetic AT. The receptors were expressed in CHO cells and tested in an aequorin-based bioluminescent calcium assay. Data are shown as relative fluorescent intensities (mean ± S.E.). (E) RT-PCR analyses of BNGR-A5, -A16, -A19, and -A22, with rpL3 as an experimental control. Brain (lane 1), CNS (lane 2), foregut (lane 3), anterior, middle and posterior midgut (lanes 4, 5, and 6), hindgut (lane 7), Malpighian tubules (lane 8), fat body (lane 9), silk gland (lane 10), testis (lane 11), ovary (lane 12), and hemocyte (lane 13).

To validate the target organ of AT and GSRYa-1 and -2 in *B*. *mori* larvae, we analyzed the expression sites of receptors for AT and GSRYa-1 and -2. We, first, screened for the GSRYa-1 and -2 receptors by Ca^2+^-imaging technique from the members in the specific clade of BNGRs for the ligands with structural similarities at their C-terminal RF-amide sequences [32], which include receptors for the extended RF-amide peptides [33]; Bm-sNPFR, and Bm-TRPR (Fig 3). Of six BNGRs (BNGR-A19, -A22, -A23, -A24, -A32, and -A33), BNGR-A19 and -A22 expressed in HEK293 cells showed increased levels of intracellular Ca^2+^ after exposure to GSRYa-1 and -2 with different affinities (Fig 4A). BNGR-A19 responded to GSRYa-1 and -2 in a dose-response manner, with a higher affinity to GSRYa-1 than that to GSRYa-2 (Fig 4B, 4C and 4E). BNGR-A22 showed much weaker responses to GSRYa-1 and -2 than BNGR-A19 (Fig 4B, 4C and 4E). The different affinities of BNGR-A19 and -A22 to GSRYa-1 and -2 indicated that BNGR-A19 functions predominantly as the receptor for GSRYa-1 and -2 in *B*. *mori*. Although the previous study reported that the AT receptor is assigned to be BNGR-A16 in Ca^2+^-imaging technique using HEK293 cell [32] (Fig 3), we newly validated the response of BNGR-A5 (Fig 3 an arrow) as well as BNGR-A16 to AT by an aequorin-based bioluminescent calcium assay in CHO cells. BNGR-A5 showed much higher affinity to AT than BNGR-A16 (Fig 4D and 4E). This high response of BNGR-A5 to AT indicated that BNGR-A5 functions predominantly as the receptor for AT in *B*. *mori*. RT-PCR revealed that GSRYa-1 and -2 receptors (BNGR-A19 and -A22) were ubiquitously expressed in the larval body (Fig 4F). In contrast, AT receptors (BNGR-A5 and -A16) were expressed in several tissues, including the brain, CNS, intestine (foregut, midgut, and hindgut), Malpighian tubules, fat body, silk gland and reproductive tissues (Fig 4F). These results implied that larval intestine (foregut, midgut, and hindgut) is one of the target organ of AT and GSRYa-1 and -2, which participates in intestinal contraction, as followingly described.

### Effects of AT and GSRYa-1 and -2 on the spontaneous contraction of foregut and hindgut in *B*. *mori* larvae

**Fig 5:**
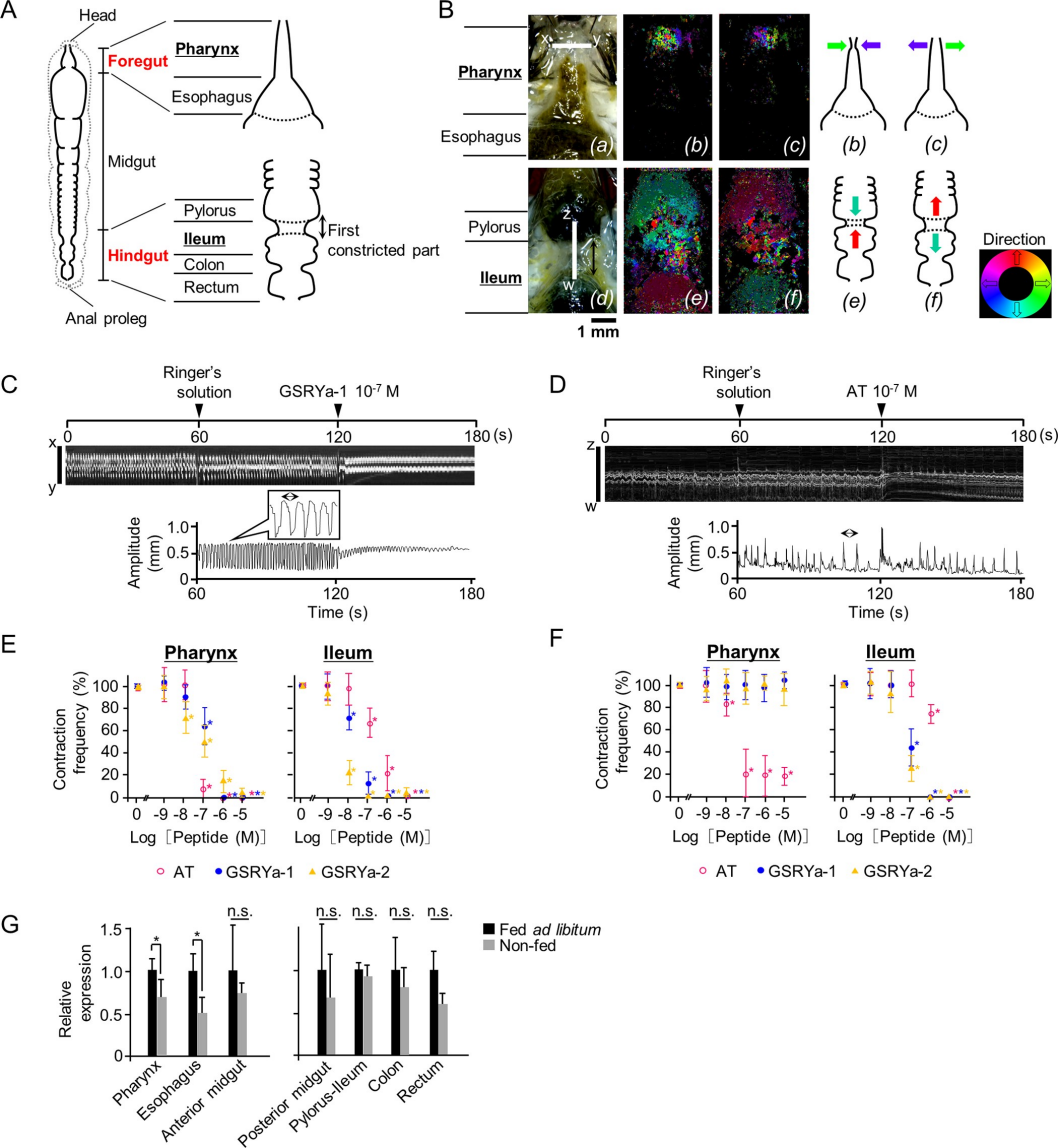
Observation of the spontaneous contraction of the foregut and hindgut in the dissected larvae. (A) A schematic diagram of larval intestine (foregut and hindgut) of *B*. *mori*. The dashed line indicates the outline of the larval body. A double-headed arrow indicates a portion of ileum. (B) *(a)* and *(d)* Pharynx and Ileum. The white axis lines (x-y for the epipharynx and z-w for the first constricted part) indicate the segment used for kymograph analysis. *(b)*, *(c)* and *(e)*, *(f)* Optic flow images of peristaltic squeeze at the pharynx and contraction at the ileum, respectively. The colored arrows in the diagrams at the right indicate the substantial direction of contractile movement of the pharynx and ileum after the “Direction” map. (C) and (D) A representative kymograph (upper) and myogram (lower) of pharynx and ileum isolated from larvae fed *ad libitum*. Arrowheads indicate the timing of exposure to *B*. *mori* Ringer’s solution at 60 s, GSRYa-1 (10^−7–6^ M) and AT (10^7−6^ M) solutions at 120 s. The waveform in the kymograph between 60 s and 180 s was converted to a myogram. The double-headed arrows indicate a single period of contraction. (E) and (F) The effects of synthetic AT and GSRYa-1 and -2 on spontaneous contraction as relative frequencies of the pharynx and ileum in larvae fed *ad libitum* (E) and non-fed (F). *B*. *mori* Ringer’s solution was administered as a vehicle control (0 M) and 10^−9^–10^−5^ M of peptide solutions were examined (%). An asterisk indicates a significant difference compared to the effect of the vehicle control by one-way ANOVA (*P* < 0.05). Data represent means ± S.D. (n = 5). (G) Quantitative-PCR analysis of BNGR-A19 in pharynx, esophagus, anterior and posterior midgut, pylorus-ileum, colon, and rectum of larvae fed *ad libitum* and non-fed. Results are represented as the ratio of BNGR-A19 to rpL3 by *C*_*t*_. An asterisk (*) indicates a significant difference compared with the larvae fed *ad libitum* in each tissue by Student’s *t*-test (*P* < 0.05). n.s. indicates no significant difference between two conditions. The data represent means + S.D. (n = 5).

Because AT and GSRYamide were the predominant brain-gut peptides in the midgut that modulated feeding initiation, we next examined the effects of these peptides on intestinal motility, particularly on the spontaneous contraction of the foregut and the hindgut [24]. In the isolated foregut of *B*. *mori* larvae, “peristaltic squeeze” was remarkably observed at the epipharynx within the pharynx, whereas little contraction was observed in the esophagus (Fig 5A and 5B*(a)–(c)*). We, then, measured the movement of the x-y axis (Fig 5B*(a)*) by kymograph analysis followed by conversion to myogram (Fig 5C). In the hindgut, predominant intestinal contraction was observed at the first constricted part within the ileum (Fig 5A and 5B*(d)–(f)*). Therefore, the anteroposterior axis over the first constricted part was analyzed (z-w in Fig 5B*(d)* and 5D). Exposure of intestines isolated from the larvae fed *ad libitum* to AT and GSRYa-1 and -2 decreased relative frequency of contraction at the pharynx and ileum (Fig 5E) (S1 Movie and S2 Movie). Similarly, exposure of non-fed larvae to AT also decreased the frequency in the pharynx and ileum (Fig 5F). By contrast, exposure of the ileum in the intestines isolated from non-fed larvae to GSRYa-1 and -2 decreased contraction in a dose-response manner (Fig 5F right), whereas no changes were observed in the pharynx (Fig 5F left).

To confirm the change of sensitivity in the pharynx (including the slightly moving hypopharynx) (Fig 5B *(b)* and *(c)*), we measured the expression level of BNGR-A19 as a predominant receptor of GSRYa-1 and -2 in order to address the different responses to GSRYa-1 and -2 between fed *ad libitum* and non-fed larvae. Quantitative-PCR revealed that BNGR-A19 expression in the hindgut (pylorus-ileum, colon, and rectum) did not fluctuate according to the feeding state (Fig 5G right), whereas a significant decrease in BNGR-A19 expression was observed in the foregut (pharynx and esophagus) (Fig 5G left).

### Effects of AT and GSRYa-1 and -2 on the esophageal contraction in *B*. *mori* larvae

**Fig 6:**
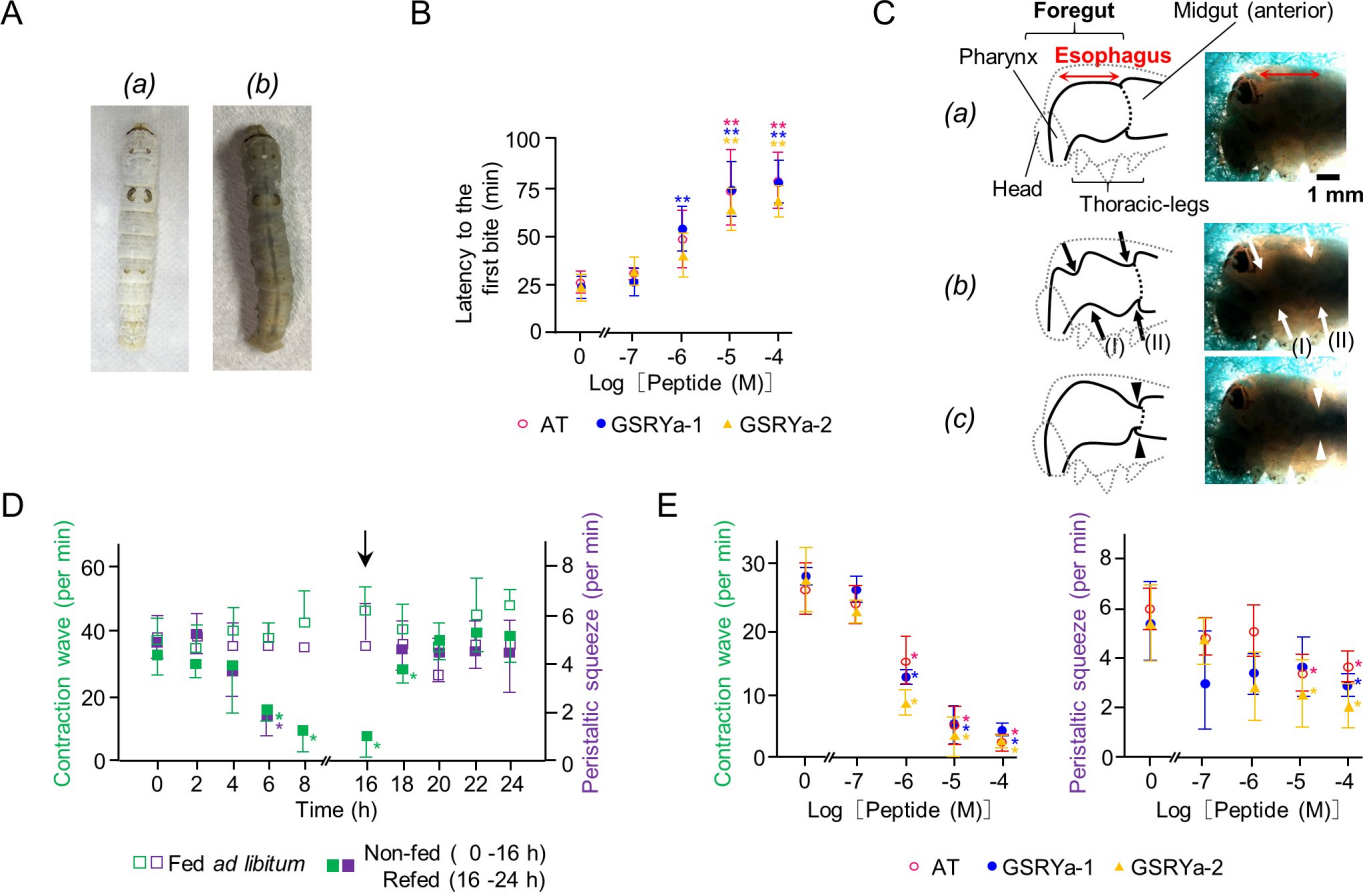
Observation of esophageal contraction in intact larvae. (A) Pictures of normal *(a)* and transparent larvae *(b)*. (B) Effects of synthetic AT (open circles), GSRYa-1 (closed circles), and GSRYa-2 (closed triangles) on the latency to the first bite. Asterisks (**) indicate significant differences compared with vehicle-injected larvae by one-way ANOVA (*P* < 0.01). Data represent means ± S.D. (n = 3). (C) Schematic diagrams and pictures of larval thorax under transmitted light. The shadowed area matches roughly with the foregut and anterior midgut; *(a)* The positions of the foregut (pharynx and esophagus) and midgut (anterior) in the figure; *(b)* Forward contraction wave: continuous contraction waves (35–45 waves/min) from the junction of the esophagus and midgut toward the pharynx (arrows). The period of a single wave (between arrow (I) and arrow (II)) was approximately 1.3–1.7 seconds; *(c)* Peristaltic squeeze: the strong and intermittent contraction (5–6 pulses/min) at the junction of the esophagus (arrowheads). (D) Fluctuations of forward contraction wave and peristaltic squeeze per minute in the two different feeding states; fed *ad libitum* (open squares), non-fed for 16 h and subsequently refed for 8 h (closed squares). An arrow indicates the timing of refeeding. An asterisk (*) indicates a significant difference compared with the larvae fed *ad libitum* by one-way ANOVA (*P* < 0.05). Data represent means ± S.D. (n = 5). (E) Effects of synthetic AT (open circles), GSRYa-1 (closed circles), and GSRYa-2 (closed triangles) on forward contraction wave and peristaltic squeeze in the larvae fed *ad libitum*. Distilled water was used as a vehicle control (0 M) and 10^−7^–10^−4^ M of peptide solution were dorsolaterally injected. Asterisks (*) indicate significant differences compared with vehicle-injected larvae by one-way ANOVA (*P* < 0.05). Data represent means ± S.D. (n = 5).

To observe intestinal contraction without opening the larval body, we prepared transparent larvae (Fig 6A*(b)*) [28] that exhibited similar inhibitory effects by injection of AT and GSRYa-1 and -2 (Fig 6B) compared to those of normal larvae (Figs 1D and 6A *(a)*). In the cephalic and thoracic parts of transparent larvae, two different contractions were observed at the esophagus [24]; a continuous “forward contraction wave” (35–45 waves/min) from the junction between the esophagus and the midgut toward the pharynx (Fig 6C *(a)* and *(b)*), and a strong and intermittent “peristaltic squeeze” (5–6 pulses/min) at the esophagus junction (Fig 6C *(a)* and *(c)*) (S3 Movie). Both contractions were observed as constitutively continuous contractions in the larvae fed *ad libitum* through 24 h (Fig 6D; open squares). However, in the non-fed larvae, these contractions gradually decreased and increased after refeeding subsequently (Fig 6D; closed squares). Injection of AT and GSRYa-1 and -2 to these transparent larvae decreased numbers of forward contraction waves and peristaltic squeezes in a dose-response manner (Fig 6E) (S4 Movie), as observed in the non-fed larvae before peptide injection (Fig 6D; closed squares).

Taken together, AT and GSRYa-1 and -2 function as feeding inhibitory peptides by modulating intestinal contraction accompanied by the feeding state transition, eventually influencing feeding termination. In addition, the feeding state changed the sensitivity of the pharynx to GSRYa-1 and -2 by changing the expression of its receptor.

## Discussion

### Identification of AT and GSRYa-1 and -2 as feeding inhibitory “brain-gut peptides” from *B*. *mori* larvae

AT was originally isolated from extracts of heads of pharate adult tobacco hornworm, *Manduca sexta* as an allatoregulatory peptide [42]. Immunohistochemistry has demonstrated the presence of AT-expressing cells in the brain, the CNS, and the midgut in *B*. *mori* [20, 43]. Although the characterization of intestinal function has not been elucidated in *B*. *mori*, the current study revealed the feeding regulatory function of AT by modulating intestinal contraction, which is a function, distinctive from allatoregulatory roles [32, 44]. Consistently, it is possible in other insect species that AT can modulate feeding motivation by its pleiotropic physiological and developmental functions [45–47] including myoregulatory activities on the foregut [25, 26, 48], midgut [27, 49] and hindgut [50, 51].

A database search showed that GSRYa-1 and -2 are orthologs of the recently discovered insect RYamide (S3 Fig). Peptides having C-terminal RYamide sequences constitute a neuropeptide Y (NPY) family, which is conserved both in vertebrates and invertebrate. As NPY has been assigned to a neuropeptide contributing to feeding behaviors in mice [52], the NPY homolog, NPF is known to regulate larval feeding behavior in *D*. *melanogaster* [53, 54]. Insect RYamide was reported to suppress feeding motivation in proboscis extension response tests for measuring feeding sensitivity when RYamide was administered to the flies, *D*. *melanogaster* [29] and *Phormia regina* [55]. The results of the present study (Figs 1D and 6B) are consistent with the context in the previous reports that RYamide functions as a feeding inhibitory peptide. In addition, GSRYamide was expressed in the reproductive organs, especially in the testis (Fig 2B). Such expression pattern of any bioactive peptides in the reproductive tissues as that of GSRYamide expression may indicate the parental function of the bioactive peptides during reproduction and development.

The myoinhibitory activities of AT and GSRYa-1 and -2 in the foregut (pharynx and esophagus) and hindgut (ileum) (Figs 5E, 5F and 6E) seem to be linked to the delay in feeding initiation in *B*. *mori* larvae (Figs 1D and 6B). The intestinal contraction, especially in the esophagus, was gradually weakened by the continuous non-feeding (quiescent) mode (Fig 6D), indicating that these peptides contribute to the shift of the larval feeding state from the feeding to non-feeding (quiescent) mode. In this study, AT showed inhibitory effect on intestinal contractions, whereas AT in several other Lepidopteran species show a stimulatory effect as described above [25, 26, 48, 51]. To know the opposite effects of AT on contractive regulation among the lepidopteran species, detail of the target for AT should be addressed. In fact, similar opposite effect in feeding behavior by a neuropeptide between species has been previously observed. For example, sNPF stimulates food intake and body weight gain in *D*. *melanogaster* [56] and has feeding acceleratory effect in *B*. *mori* [16], whereas an opposite behavioral effect, an inhibitory effect on food intake, is observed in *Schistocerca*. *gregaria*, [57]. In the non-fed *B*. *mori* larvae, exposure of the pharynx to GSRYa-1 and -2 did not decrease its contractions (Fig 5F left), indicating that the feeding state transition by GSRYa-1 and -2 (Figs 1D and 6B) is likely to be induced only by the inhibition of hindgut (ileum) contraction. Although few studies have focused on these kinds of effects of different peptides in the species level or in the individual level, this intriguing effect of AT and GSRYa-1 and -2 may be crucial in determining the unknown pleiotropic function of these peptides. In addition, the inhibition after exposure to AT and GSRYa-1, 2 did not resume even after washing with the vehicle without the ligands.

**Fig 7:**
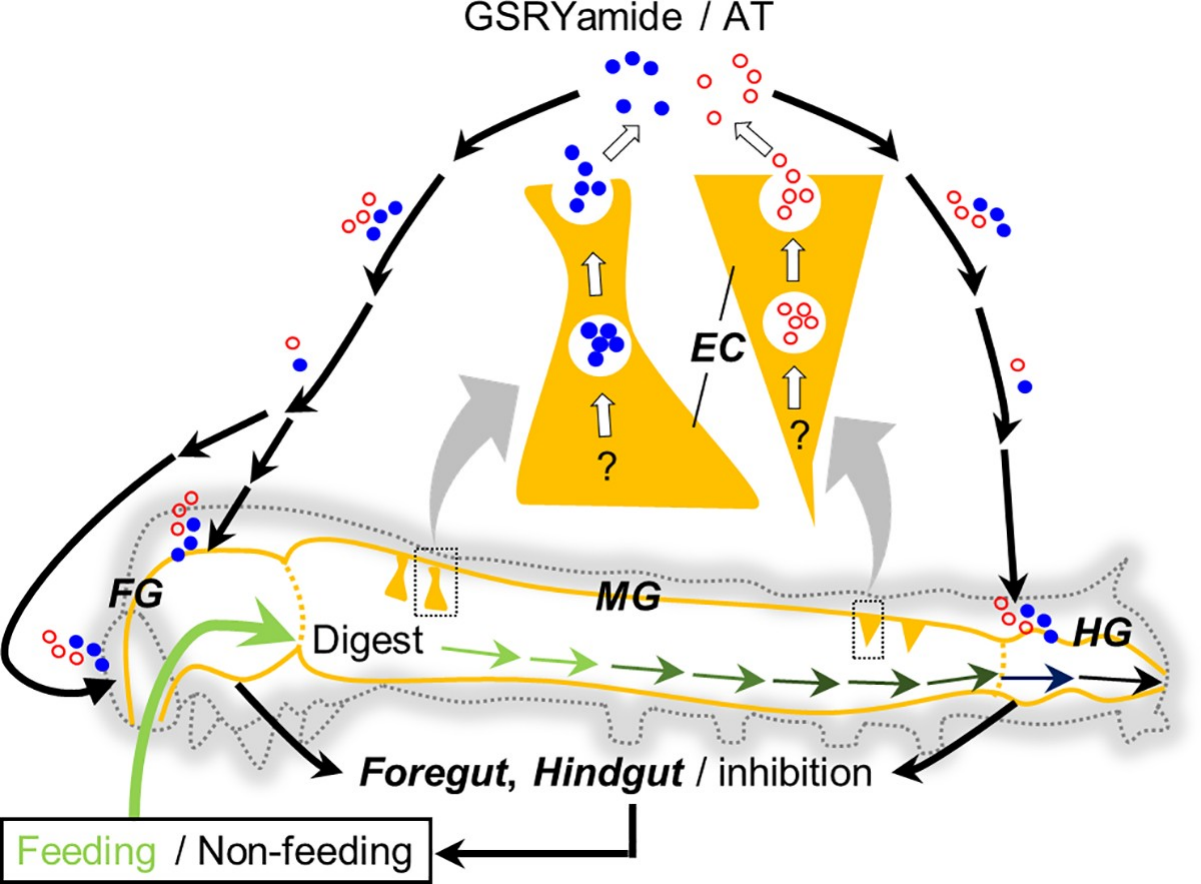
Proposed regulatory model of feeding behavior in *B*. *mori* larvae. AT and GSRYamide are secreted from the posterior and anterior midgut EEs. After circulating in the hemolymph, these peptides act directly on the foregut and hindgut to inhibit intestinal contraction. This myoinhibitory effect induces a larval feeding state transition from the feeding to the non-feeding (quiescent) mode, eventually influencing feeding termination. *EEs*: enteroendocrine cells, *FG*: foregut, *MG*: midgut, *HG*: hindgut.

In *B*. *mori*, 40 BNGRs are expressed in different tissues in distinct expression patterns [32]. Among the receptors, several receptors show ubiquitous expression patterns as observed in four BNGRs in this study (BNGR-A5, -A16, -A19, and -A22), indicating that GSRYa-1 and -2 may have pleiotropic functions as well as AT (Fig 4F). In this study, the expression patterns of those receptors in the peristaltic intestine (Fig 4F) covered the area where contraction was influenced by both AT and GSRYa-1 and -2 (Fig 7). Because different sensitivities of the pharynx to AT and GSRYa-1 and -2 were observed in the non-fed larvae (Fig 5F left), the feeding state can also be shifted or modified by altered expression levels of GSRYa-1 and -2 receptors. During the states of hunger and subsequent lack of nutrition, extended feeding duration to ingest more food might occur in the pharynx and esophagus as a machinery to food entry the food by decreasing the sensitivities to feeding inhibitory GSRYa-1 and -2 following the reduced expression of its receptor (Fig 5G).

### Model of feeding regulatory mechanism via control of intestinal contraction in *B*. *mori* larvae

Previous ultrastructural studies have demonstrated that exocytosis occurs at both the basal and lateral surfaces of insect midgut EEs, depicting the possibilities of secretion of biologically active compounds [58, 59]. AT-producing EEs (Fig 2C and 2D) and GSRYamide-producing EEs (Fig 2E and 2F) also have the potential to deliver secretory granules containing peptidyl factors to the hemocoel side through a cellular membrane [60]. Those EEs have similar cellular morphology to EEs releasing other arthropod neuropeptide CCAP (Crustacean cardioactive peptide) observed in the American cockroach, *Periplaneta americana* [61]. CCAP has multiple roles, including gut contraction [61] as observed for AT and GSRYamide in the present study. Similarly, the presence of EEs secreting allatoregulatory peptides has also been reported in Dipteran and Hemipteran species [62–65]. In other studies, AT and allatostatin (AST) regulate spontaneous contraction of the foregut of *Helicoverpa armigera* [25] and *Lacanobia oleracea* [26]. Furthermore, myosuppressin (leukomyosuppressin), a member of the structurally related RFamide peptide family [33], also regulates spontaneous contraction of the foregut of *Lacanobia oleracea* [66] and the hindgut of *Leucophaea maderae* [13]. In fact, we have observed several fractions with weak inhibitory activities during the purification steps, which may correspond to the previous reports that those allatoregulatory peptides such as AST and FGLamide are present in the midgut and modulate the gut contraction. Taken together, midgut-derived AT and GSRYamide can contribute predominantly to the regulation of feeding behavior by modulating intestinal contraction as currently depicted in a model of “feeding-termination loop” (Fig 7). First, AT and GSRYamide are secreted from the posterior and anterior midgut EEs, respectively. Once these secreted peptides circulate in the hemolymph, they act directly on the foregut and hindgut to inhibit intestinal contraction. These myoinhibitory effects induce the stagnation of the ingested food in the foregut and fecal passage through the hindgut, accompanied by a larval feeding state transition from the feeding to the non-feeding (quiescent) mode. Consequently, these peptides appear to induce feeding termination. A previous study demonstrated that the arrest of pharynx movement by the stagnation of the ingested food induces the quiescent mode of feeding in *B*. *mori* [67].

In many insect species, foregut muscular cells are innervated from the brain and frontal ganglion [68] within a short neural circuit involving the stomatogastric central pattern generator [5, 69]. Many neuropeptides, such as AST-A, RFamide, sNPF and TRPs including AT, have been observed in the stomatogastric nervous system (SNS) [5]. In particular, AT can contribute to the modulation of feeding via SNS by controlling spontaneous foregut contractions in many insect species, including *D*. *melanogaster*, and *M*. *sexta* [70–72]. The interaction of those neuropeptides, such as myoactive peptides that regulate intestinal contraction, may play an important role in the shift of the larval feeding state by harmonization of those neuropeptides. In *B*. *mori* larvae, pharynx movements for swallowing food are linked by a neuron projecting from brain and frontal ganglion [73]. In addition, AT-driving contractions of the foregut and hindgut may be produced by neurons innervating from these parts of the intestine [20]. The expression of *B*. *mori* sNPF and TRPs, which have feeding acceleratory effects [16], and their receptor BNGRs (BNGR-A10, -24, and -A32) were confirmed in the brain and CNS [34, 40, 74]. Therefore, the expression of AT, GSRYamide and their receptor BNGRs (BNGR-A5, -16, -A19, and -A22) in the brain and CNS (Figs 2B and 4F) implies that AT and GSRYa-1 and -2 can function as neurotransmitters and interact with sNPF or TRPs to control intestinal contractions in *B*. *mori* larvae.

Neuropeptides released from nerve terminal also function as endocrine factors in addition to as the neurotransmitters [75, 76]. AT produced from CNS travels through ventral nerve cord and gets released into the hemolymph to modulate the intestinal contraction. However, the number of AT-producing EEs in the whole midgut seems to be much higher than that in the CNS (Fig 2C) [20, 77]. Therefore, the main source of AT released into the hemolymph is probably midgut EEs. In addition, the previous report demonstrates that GSRYamide is produced only in the interneurons within CNS in this species [19]. Therefore, midgut EEs are the only possible source of GSRYamide in the hemolymph. Our proposed regulatory model is more plausible for GSRYamide.

Although it remains to be elucidated whether neurosecretory AT affects the intestinal contractions, our findings in this study suggest that hormonal AT and GSRYamide secreted from the midgut to the hemolymph can also contribute to the physiological functions, including regulation in intestinal contraction of *B*. *mori*. In addition, our “feeding-termination loop” model provides a response to acute EE-derived feeding regulatory signals without mediation via the brain or CNS, allowing for a smooth feeding state transition.

## Supporting information

S1 Fig MALDI-TOF mass analyses of the final step purified fractions A1 and B3. (A) MALDI-TOF mass spectrum of A1 fraction (Fig 1A). Monoisotopic ion peak ([M+H]^+^) at *m*/*z* 1486.9 consistent with the H^+^ adduct of uncharged AT is labeled. (B) MALDI-TOF mass spectrum of B3 fraction (Fig 1B). Monoisotopic ion peak ([M+H]^+^) at *m*/*z* 1999.9 consistent with the H^+^ adduct of uncharged GSRYa-1 is labeled.
(TIF)

S2 Fig cDNA and deduced amino acid sequences encoding GSRYa-1 and -2. The deduced mature GSRYa-1 and -2 are underlined by a bold line and a dashed line, respectively. Arrows (GSRYa-Fw and GSRYa-Rv) represent the forward and reverse primer sites for RT-PCR and *in situ* hybridization. The stop codon is indicated by an asterisk.
(TIF)

S3 Fig Alignment of the amino acid sequences among *B*. *mori* GSRYamides and other insect RYamides. By BLAST (blastn, tblastn and Blastp) search in the GenBank database, similar sequences to GSRYa-1 and -2 were obtained. *B*. *mori* GSRYa-1 and -2, RYa-1 and -2 of *D*. *melanogaster* [29], *T*. *castaneum* and *Aedes aegypti* [30] are aligned. Residues identical between peptides are shaded.
(TIF)

S1 Movie The contraction of pharynx after exposure to *B*. *mori* Ringer’s solution and GSRYa-1. *B*. *mori* Ringer’s solution and GSRYa-1 (10^−6^ M) solution were exposed at 60 s and 120 s in Fig 5C (Arrowheads). After exposure to GSRYa-1, the contraction of pharynx was inhibited, while exposure of *B*. *mori* Ringer’s solution had little effect on the contraction.
(MP4)

S2 Movie The contraction of ileum after exposure to *B*. *mori* Ringer’s solution and AT. *B*. *mori* Ringer’s solution and AT (10^−6^ M) solution were exposed at 60 s and 120 s in Fig 5D (Arrowheads). After exposure to AT, the contraction of ileum was inhibited, while exposure of *B*. *mori* Ringer’s solution had little effect on the contraction.
(MP4)

S3 Movie The esophageal contraction. Forward contraction wave (Fig 6C *(b)*) and peristaltic squeeze (Fig 6C *(c)*) were visualized under the transmitted light in the transparent larvae.
(MP4)

S4 Movie The esophageal contraction before and after injection of AT. AT (10^−5^ M) solution was injected dorsolaterally into the hemolymph. After injection of AT, the forward contraction wave and peristaltic squeeze were inhibited.
(MP4)
