# Supplementary figures and images for: Enteroendocrine peptides regulate feeding behavior via controlling intestinal contraction of the silkworm Bombyx mori
Source: PLoS One. 2019 Jul 1;14(7):e0219050. doi: 10.1371/journal.pone.0219050 (PMC6602202; doi:10.1371/journal.pone.0219050)

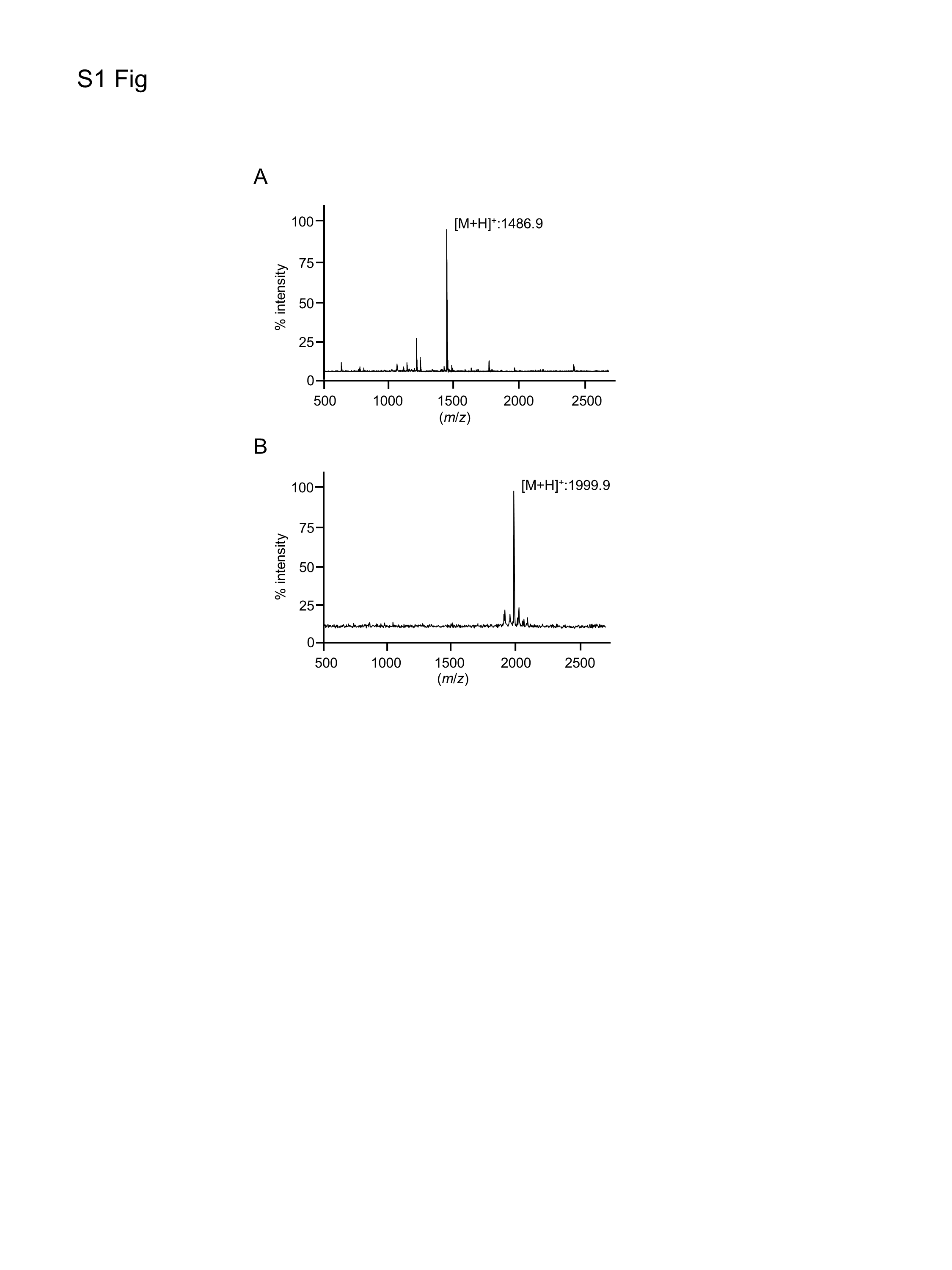

Supplement: S1 Fig — (A) MALDI-TOF mass spectrum of A1 fraction (Fig 1A). Monoisotopic ion peak ([M+H]+) at m/z 1486.9 consistent with the H+ adduct of uncharged AT is labeled. (B) MALDI-TOF mass spectrum of B3 fraction (Fig 1B). Monoisotopic ion peak ([M+H]+) at m/z 1999.9 consistent with the H+ adduct of uncharged GSRYa-1 is labeled. (TIF) [file pone.0219050.s001.tif]

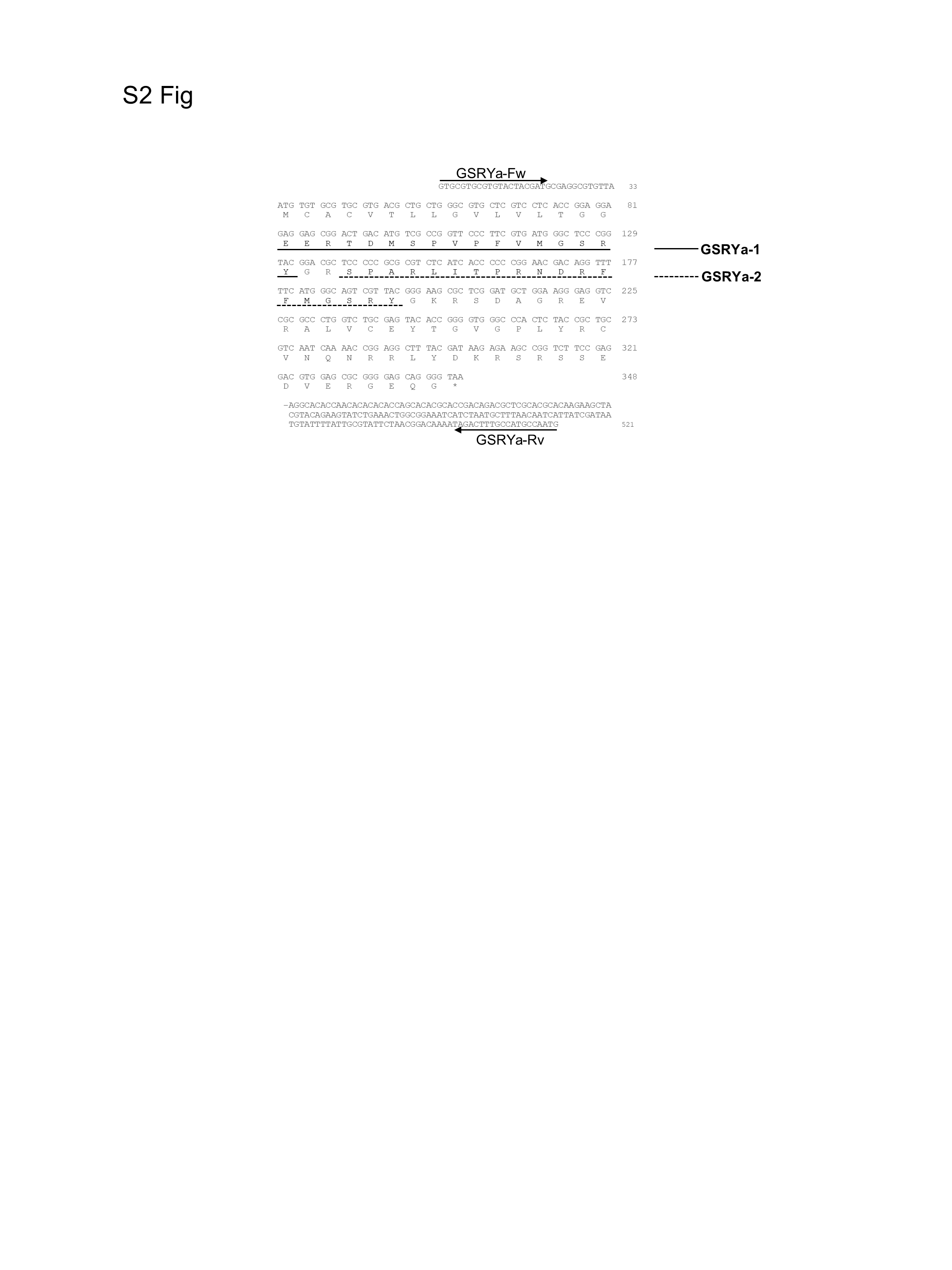

Supplement: S2 Fig — The deduced mature GSRYa-1 and -2 are underlined by a bold line and a dashed line, respectively. Arrows (GSRYa-Fw and GSRYa-Rv) represent the forward and reverse primer sites for RT-PCR and in situ hybridization. The stop codon is indicated by an asterisk. (TIF) [file pone.0219050.s002.tif]

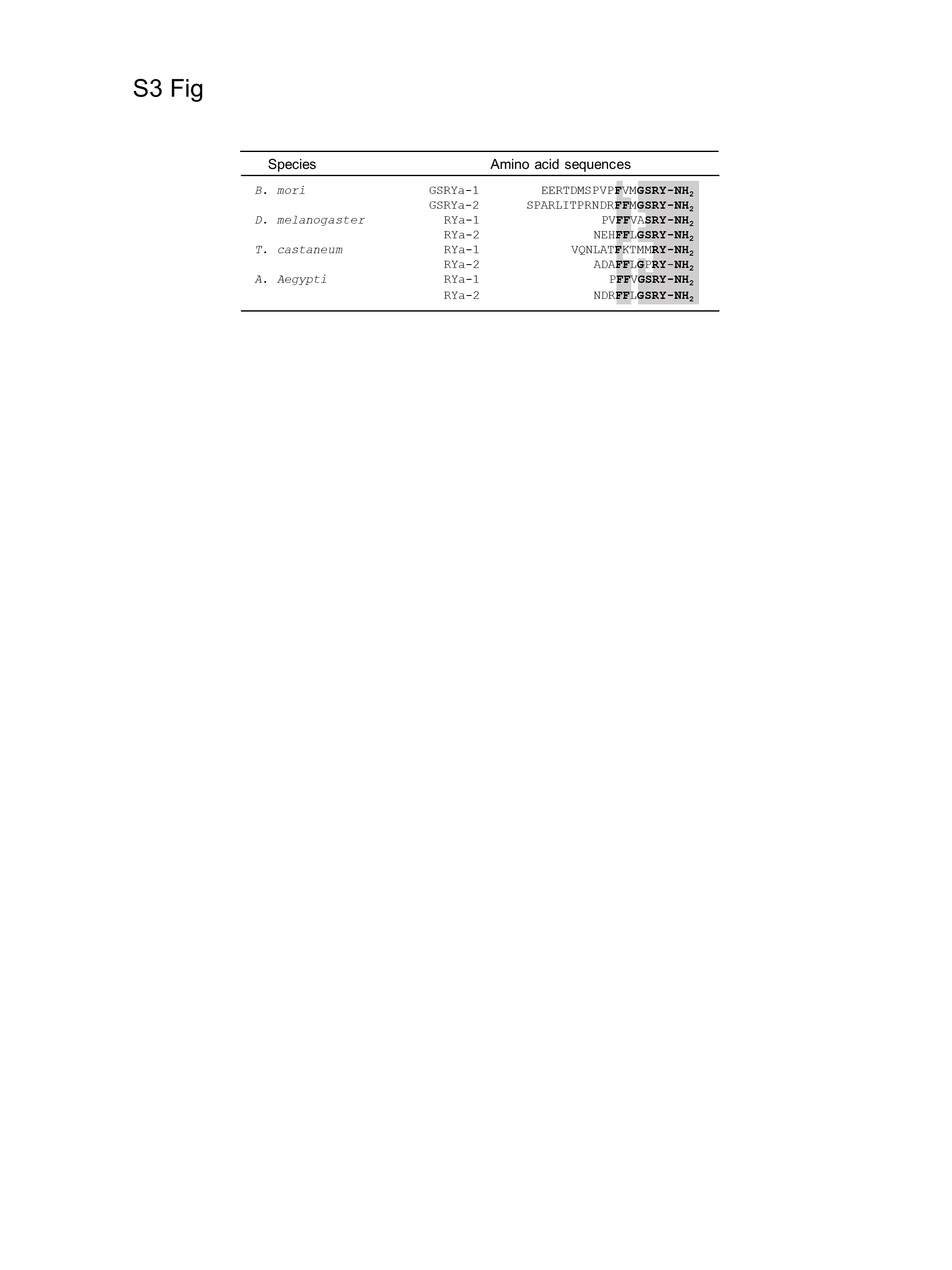

Supplement: S3 Fig — By BLAST (blastn, tblastn and Blastp) search in the GenBank database, similar sequences to GSRYa-1 and -2 were obtained. B. mori GSRYa-1 and -2, RYa-1 and -2 of D. melanogaster [29], T. castaneum and Aedes aegypti [30] are aligned. Residues identical between peptides are shaded. (TIF) [file pone.0219050.s003.tif]
